# Subcutaneous *Eikenella corrodens*, *Actinomyces* sp., and *α*-Hemolytic *Streptococcus* Abscess of the Thigh following a Vitamin B12 Injection

**DOI:** 10.1155/2018/4650637

**Published:** 2018-04-11

**Authors:** Micheal G. Adondakis, John G. Skedros, Bert K. Lopansri, Stephen C. Merrell

**Affiliations:** ^1^Utah Orthopaedic Specialists, Salt Lake City, UT, USA; ^2^University of Utah Department of Orthopaedic Surgery, Salt Lake City, UT, USA; ^3^Division of Infectious Diseases and Clinical Epidemiology, Intermountain Healthcare, Intermountain Medical Center and LDS Hospital, Salt Lake City, UT, USA; ^4^Department of Medicine, University of Utah, Salt Lake City, UT, USA; ^5^Department of Family Medicine, Intermountain Medical Group, Bountiful, UT, USA

## Abstract

This case report describes a 38-year-old female presenting with a thigh abscess caused by *Eikenella corrodens*, *Actinomyces* sp., and α-hemolytic *Streptococcus* following an intramuscular vitamin B12 injection administered at an outpatient clinic. After failure to improve clinically with intravenous daptomycin and after visualization of the abscess with gas bubbles on CT scan, she was taken to the operating room for three separate surgical irrigation and debridement procedures. Treatment also included intravenous ampicillin/sulbactam followed by oral amoxicillin/clavulanic acid therapy. She remained symptom free and without infection at nine months following hospitalization. It was suspected that poor hygiene played a role in the infection, but a definitive cause was not identified.

## 1. Introduction

*Eikenella corrodens* (*E. corrodens*) is a facultative anaerobic bacteria normally found in the oral flora. It has been reported to rarely cause infections following bite wounds and oral and GI surgery and has a predilection toward intravenous drug users, diabetic patients, and patients who are otherwise immunocompromised [1–3]. Abscess formation caused by this organism, often with one or two additional bacteria, has been reported to occur most often in the head and neck region but also less commonly in other parts of the body [4, 5]. Here, we report a unique case of a thigh abscess following an intramuscular vitamin B12 injection, and cultures grew *E. corrodens*, *Actinomyces* sp., and α-hemolytic *Streptococcus*. The infection was successfully treated with multiple surgical debridements and antibiotic therapy.

## 2. Case Report

The patient is a 38-year-old female who presented to our hospital's emergency department (ED) with left lateral thigh pain, erythema, warmth, and swelling of two days duration. The patient believed that her left thigh pain and swelling was related to an intramuscular vitamin B12 (cyanocobalamin) injection that was administered at her primary care physician's office three days prior to presentation.

Her medical and surgical history included Graves' disease, paroxysmal supraventricular tachycardia, schizoaffective disorder, abdominoplasty, and laparoscopy with lysis of pelvic adhesions. Outpatient medications included trazodone, quetiapine, citalopram, and levothyroxine. She smoked half a pack of cigarettes per day, drank alcoholic beverages 2-3 times per week, and had a history of methamphetamine use, smoking, and injecting. She reported that her last use of any illicit drugs was 17 months before this ED visit.

On examination, there was a 12 cm diameter area of induration and erythema over the lateral aspect of her left upper thigh. This area was also warm and exquisitely tender to palpation, and there was pain with abduction and axial loading of hip. Her laboratory studies showed increased white blood cell (WBC) count of 12.4 (normal is ≤10.9), C-reactive protein (CRP) of 10.4 (normal is ≤0.9), and unremarkable erythrocyte sedimentation rate (ESR) of 15 (normal is ≤10). Blood and urine testing for drugs (including illicit substances) was not done. Fluoroscopic-guided left hip joint aspiration revealed no fluid, and radiographs did not reveal any abnormality. She was given one intravenous dose of daptomycin. She returned to the ED on the following day for a scheduled second dose of daptomycin. At that visit, she reported no change in her symptoms.

**Figure 1:**
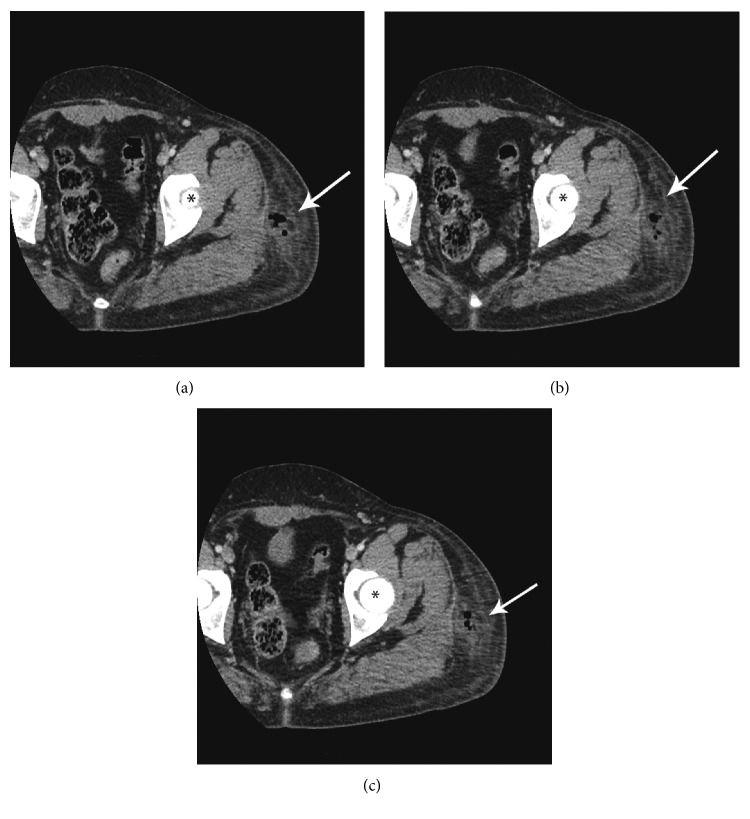
(a, b, c) Three sequential transverse section CT scan images showing the abscess with gas bubbles (arrows); the femoral head is indicated with an asterisk.

The following day, she returned to the ED. Examination showed that the erythema had spread. All infection markers had also increased: WBC count was 14.9, CRP was 23.0, and ESR was 39. A CT scan showed a 3.5 × 2.5 cm collection of gas with fluid within the subcutaneous fat over the upper thigh near the lateral greater trochanter region (Figure 1). She was admitted for intravenous antibiotic therapy with cefazolin and vancomycin and for surgical irrigation and debridement.

Irrigation and debridement was performed on the date of hospital admission. There was 10 milliliters of malodorous pale green pus with gas bubbles. There was no evidence of necrotizing fasciitis, myonecrosis, or deep sinus tracts. A conventional wound sponge and vacuum was placed. A second irrigation and debridement was done three days later, and a new would vacuum sponge was placed.

Cultures grew *E. corrodens*, *Actinomyces* sp., and α-hemolytic *Streptococcus*. She initially received intravenous vancomycin and cefazolin which were changed to intravenous ampicillin/sulbactam once culture results were available. She continued to improve, and on the sixth hospital day, she underwent a third irrigation and debridement with primary closure of the wound over two suction drains. The patient was discharged to home the following day on intravenous ampicillin-sulbactam for five days and then oral amoxicillin-clavulanic acid for an additional 14 days. She had complete resolution of symptoms and remained free of infection at nine months after her final surgery.

Our patient had received a standard 1000 mcg dose of vitamin B12 from a single-dose vial. The Utah State Health Department and the USA Federal Drug Administration (FDA) were contacted regarding the case, and the remaining vials from the vitamin B12 lot were quarantined. The FDA recommended against culturing the remaining vials because a false positive result would be difficult to interpret and because no other infections from that lot had been reported either locally or nationally. Our patient had previously received a vitamin B12 injection from the same lot to the opposite hip without incident. The injection that preceded the infection had been administered by a board-certified medical assistant trained in intramuscular injections. She reported to have cleaned the skin with an alcohol wipe, to have waited approximately 15 seconds for the skin to dry before injecting the dose, and to have covered the insertion site with a Band-aid. Our patient does not remember her waiting for the skin to dry, nor a Band-aid being placed over the insertion site, and believes that the needle for the injection was removed from its cap by biting the cap. It is also important to note that our patient does have a pet dog, who may have introduced the oral flora to the skin either before or after the injection.

## 3. Discussion

This is a novel case of an abscess of the superficial tissues of the thigh that grew *E. corrodens* and two additional organisms several days after administration of a vitamin B12 injection in an outpatient setting. The most unusual aspect of this case was the growth of *E. corrodens*, which is a gram-negative facultative anaerobic bacillus that is normally found in the oral flora and oropharyngeal mucosa as well as in the GI tract [6]. Infections from this organism with abscess formation most often occur in the head and neck region including the thyroid gland and are rare in other soft tissues, bones, and joints [5]. However, a few cases of osteomyelitis and septic arthritis of the hip due to *E. corrodens* have been reported following oral bite wounds and fork injuries, punctate lacerations of the lower and upper extremities, dental and GI procedures, or with idiopathic etiology [1, 2, 5].

When *E. corrodens* causes an infection, it is usually a polymicrobial infection and very often associated with *Streptococcus* organisms (as seen in our case) at rates of up to 50% of these infections in children and approximately 70% in adults [7, 8]. As in our case, *E. corrodens* infections associated with *Actinomyces* have been reported [9–11]. These organisms that are typically found along with *E. corrodens* are usually in the normal oral flora, and thus it stands to reason that these would be typically associated with head and neck infections and in injection drug users (probably because of the tendency to lick their needles) [12, 13]. However, in our patient, it is very unusual that these organisms would cause an abscess because she had no oral surgeries or other procedures that could cause hematogenous spread of this organism, and her injection drug use was remote. Though a relapse in her intravenous drug use is possible, injecting drugs of abuse into the skin at the hip is not a common practice. Contamination via the skin seems to be the most plausible explanation for her thigh abscess, which was also likely placed in the subcutaneous tissues and not intramuscular as was intended. It is possible that not enough time was given for the alcohol to disinfect the skin before the injection occurred. It is also possible that our patient's dog introduced the bacteria by licking the site. Lastly, the needle could have touched the mucus membranes of our medical assistant prior to the injection, introducing the oral flora as the needle may have been removed from its cap by biting the cap.

The development of limb abscesses caused by *E. corrodens* that requires both surgical and antibiotic treatment is rare. For example, Zhiyong et al. [8] reported a case of a 35-year-old Chinese female patient who was described as healthy and developed a thigh abscess from which *E. corrodens* and *S. intermedius* were grown. Similar to our case, the cause of that infection was not determined. However, unlike our patient, their patient denied having a known skin puncture and other episodes or instances that could have violated the skin surface. Following failed prior therapy with intravenous cefazolin, vancomycin, and clindamycin, her polymicrobial infection was successfully treated with surgical debridement along with intravenous ceftriaxone therapy.

There is also a report of two adolescent females who developed abscesses with *E. corrodens* positive cultures [3]. The first patient developed a finger abscess from chronic finger biting, which resulted in a complex felon. Initial treatment with oral cephalexin failed. The infection was then successfully treated with incision and drainage and intravenous penicillin-G and gentamicin after culture sensitivities were obtained. The second patient had poorly controlled diabetes for five years. She developed an acute thigh abscess at an insulin injection site that resolved after surgical debridement and intravenous nafcillin followed by oral cephalexin after discharge from the hospital. The infection in this latter case demonstrates that *E. corrodens* can be present on the skin in the area of the thigh.

*E. corrodens* is also known to be relatively more commonly associated with infections in injection drug users, perhaps due to the tendency of users to lick needles. In a retrospective trial studying 59 abscesses of the upper extremity in injection drug users, *E. corrodens* was found to be among the most common causative organisms [14]. Armstrong and Fisher [4] reported a case of an upper extremity abscess in an injection drug user that required surgical treatment and intravenous antibiotics for infection with *E. corrodens* and other organisms found in the oral flora. To our knowledge, no prior case of abscess formation following attempted iatrogenic intramuscular vitamin B12 injection has been reported in the literature.

## 4. Conclusion

This is a case of a 38-year-old woman who developed an abscess due to *E. corrodens*, *Actinomyces* sp., and α-hemolytic *Streptococcus* following an attempted intramuscular vitamin B12 injection in the primary care setting. The polymicrobial infection was successfully treated with intravenous antibiotics and three irrigation and debridement procedures in the operating room. Though a definitive cause was not determined in this case, it is possible that bacteria were introduced from the skin after inadequate disinfection preparation with isopropyl alcohol, or from removing the cap of the needle by biting it, or from our patient's dog licking her thigh injection site.
